# RETRACTION: Acute Burns During the COVID‐19 Pandemic: A One‐Year Retrospective Study of 611 Patients at a Referral Burn Centre in Northern Iran

**DOI:** 10.1111/iwj.70254

**Published:** 2025-02-25

## Abstract

**RETRACTION:** MobayenM. , TorabiH. , ToolaroudP. B. , ToloueiM. , MoghadamA. D. , SaadatmandM. , EslamiKenarsariH. , FeizkhahA. , GhazanfariM. J. , OsujiJ. , VajargahP. G. , and KarkhahS. , “Acute Burns During the COVID‐19 Pandemic: A One‐Year Retrospective Study of 611 Patients at a Referral Burn Centre in Northern Iran,” International Wound Journal 20, no. 8 (2023): 3204–3211, 10.1111/iwj.14199.37095647 PMC10502268

The above article, published online on 24 April 2023, in Wiley Online Library (http://onlinelibrary.wiley.com/), has been retracted by agreement between the journal Editor in Chief, Professor Keith Harding; and John Wiley & Sons Ltd. A third party reported to the journal that they had found evidence of excessive self‐citations in the reference list of this article. The publisher confirmed multiple instances of redundant self‐citations. Upon further investigation, the publisher also concluded that this article was accepted solely on the basis of a compromised peer review process. In addition, the article does not include any information about the informed consent procedure for the study. In view of the clear evidence of compromised peer review, the parties agreed that the paper must be retracted. The authors disagree with the retraction.

**RETRACTION:** M. Mobayen , H. Torabi , P. B. Toolaroud , M. Tolouei , A. D. Moghadam , M. Saadatmand , H. EslamiKenarsari , A. Feizkhah , M. J. Ghazanfari , J. Osuji , P. G. Vajargah , and S. Karkhah , “Acute Burns During the COVID‐19 Pandemic: A One‐Year Retrospective Study of 611 Patients at a Referral Burn Centre in Northern Iran,” International Wound Journal 20, no. 8 (2023): 3204–3211, 10.1111/iwj.14199.37095647 PMC10502268

The above article, published online on 24 April 2023, in Wiley Online Library (http://onlinelibrary.wiley.com/), has been retracted by agreement between the journal Editor in Chief, Professor Keith Harding; and John Wiley & Sons Ltd. A third party reported to the journal that they had found evidence of excessive self‐citations in the reference list of this article. The publisher confirmed multiple instances of redundant self‐citations. Upon further investigation, the publisher also concluded that this article was accepted solely on the basis of a compromised peer review process. In addition, the article does not include any information about the informed consent procedure for the study. In view of the clear evidence of compromised peer review, the parties agreed that the paper must be retracted. The authors disagree with the retraction.

